# Implementation of a Standardized Post-Cesarean Delivery Order Set with Multimodal Combination Analgesia Reduces Inpatient Opioid Usage

**DOI:** 10.3390/jcm10010007

**Published:** 2020-12-22

**Authors:** Eran Bornstein, Gregg Husk, Erez Lenchner, Amos Grunebaum, Therese Gadomski, Cristina Zottola, Sarah Werner, Jamie S. Hirsch, Frank A. Chervenak

**Affiliations:** 1 Division of Maternal-Fetal Medicine, Department of Obstetrics and Gynecology, Lenox Hill Hospital—Northwell Health/Zucker School of Medicine, New York, NY 10075, USA; amos.grunebaum@gmail.com (A.G.); t.e.gadomski@gmail.com (T.G.); czottola@northwell.edu (C.Z.); SWerner4@northwell.edu (S.W.); fchervenak@northwell.edu (F.A.C.); 2 Department of Medical Informatics, Lenox Hill Hospital—Northwell Health/Zucker School of Medicine, New York, NY 10075, USA; Ghusk@northwell.edu; 3 Department of Biostatistics and Data Management, New York University Rory Meyers College of Nursing, New York, NY 10010, USA; erez.lenchner@gmail.com; 4 Division of Kidney Diseases and Hypertension, Department of Medicine, Donald and Barbara Zucker School of Medicine at Hofstra/Northwell, Great Neck, NY 11030, USA; jhirsch8@northwell.edu

**Keywords:** multimodal analgesics, opioid, opioid use, narcotics, post-partum pain control, post-cesarean pain control, order set

## Abstract

**Background:** Opioid use has emerged as a leading cause of death in the US. Given that 1 in 300 opioid-naive patients exposed to opioids after cesarean birth will become persistent users, hospitals should strive to limit exposure to these medications. We set out to evaluate whether transitioning to a standardized order set based on multimodal combination analgesic therapy decreases the exposure to opioids after cesarean delivery. **Methods:** Our health system’s post-cesarean pain management electronic medical record (EMR) order set was changed from standing NSAIDs (Ibuprofen 600 mg every 6 h) and additional acetaminophen and opioid medications (Oxycodone 5 mg/acetaminophen 325 mg every 3 h or Oxycodone 10 mg/acetaminophen 650 mg every 6 h for moderate and severe pain, respectively) as needed (PRN) to a multimodal combination therapy with acetaminophen (975 mg every 6 h) and NSAIDs (Ibuprofen 600 mg every 6 h) as primary analgesics and opioids PRN (Oxycodone immediate release (IR) 5 mg every 3 h for moderate to severe pain). We performed a retrospective analysis across seven hospitals comparing inpatient opioid use, administration of other analgesics, and severe pain episodes (pain score ≥ 7) between the patients who were treated before and after implementation of the multimodal order set. Chi square and Student *t*-test were used for statistical analysis with significance determined as *p* < 0.05. **Results:** A total of 12,898 cesarean births were included (8696 prior and 4202 after implementation). The multimodal order set was associated with marked decrease in the incidence of post cesarean opioid use (45.4% vs. 67.5%; *p* < 0.0001), lower average opioid dose (26.7 mg vs. 36.6 mg of oxycodone; *p* < 0.0001), and increased dose of acetaminophen (8422 mg vs. 4563 mg; *p* < 0.0001), while severe pain scores were less frequent (46.3% vs. 56.6%, *p* < 0.0001). **Conclusions:** Multimodal analgesic therapy for post-cesarean pain management reduces inpatient opioid use while improving pain control. Incorporation of a multimodal order set as a default in the EMR facilitates effective and widespread implementation on a large scale. Obstetric units should consider standardizing post-cesarean pain management orders to include routine (not PRN) multimodal combination therapy with acetaminophen and NSAIDs as primary analgesics.

## 1. Introduction

Opioid use has emerged as a leading cause of death in the United States, reaching epidemic proportions in recent years [1]. In fact, opioid-related deaths have more than tripled since the late 1990s, with an estimated 47,600 overdose deaths in 2017 accounting for 67.8% of all drug overdose deaths recorded that year [1]. The most recent data from the Centers for Disease Control and Prevention (CDC) suggest that on average 130 Americans die every day from an opioid overdose—including prescription and illicit narcotics [2]. Women have been particularly impacted, with a 5-fold increase in the incidence of opioid-related deaths over the last decade [3].

Post-surgical opioid prescriptions are widespread and are frequently the first time a patient will be exposed to opioids. Cesarean delivery is the most common inpatient surgical procedure in the United States, affecting approximately 32% of all births [4]. Most women following cesarean delivery are administered opioids for pain control, with as many as 87% of women given a prescription for opioids upon discharge home based on one study [5]. Approximately 1 in 300 opioid-naive patients exposed to opioids after cesarean birth will become persistent users. Thus, the post cesarean delivery recovery period has the potential for many opioid-naïve women to become addicted [6].

Multimodal pain therapy utilizes scheduled dosing of several different medications with varying mechanisms of action to control pain while limiting exposure to opioids and thus their potential negative consequences. The American College of Obstetricians and Gynecologists (ACOG) and other societies including the American Society of Anesthesiologists Task Force on Acute Pain Management recommend the use of multimodal analgesic therapy whenever possible for patients with acute postoperative pain [7,8,9]. Nevertheless, the data regarding the use of this modality is still accumulating and implementation of such practice is not commonplace.

The purpose of our study was to evaluate the potential impact of adopting a standardized order set based on multimodal combination analgesic therapy on both opioid usage as well as quality of pain control in the immediate post cesarean delivery period in a large healthcare system with multiple obstetrical units.

## 2. Experimental Section

This is a retrospective analysis of data on all patients who had cesarean delivery between January 2018 and December 2019 in seven hospitals in our health system that has over 30,000 combined annual births and share an enterprise electronic medical record (EMR). Data were extracted directly from the EMR, which contains all post-operative orders, medications administration data (type, dose, timing), and pain scale assessments. Information regarding medication administration and dosages as well as pain scale assessments are available on patients from admission to discharge.

In May 2019, our institution’s post-cesarean pain management order set was changed in our EMR to include a default treatment with combination of agents (multimodal) based on the recommendations from the ACOG [7]. The analysis aimed at detecting potential differences in the administration of opioids, as well as the patients’ subjective assessment of the quality of pain control following cesarean delivery before and after the implementation of the multimodal post cesarean order set.

We divided our cohort into two groups based on the date of cesarean delivery reflecting the type of post cesarean pain management order set used (old versus multimodal). The first group consisted of all cesarean deliveries in the 16 months prior to implementing the multimodal analgesic order set (between January 2018 and April 2019), i.e., ‘old’ order set. This group was compared to the group of patients who had cesarean delivery in the first eight months following the implementation of the multimodal analgesic order set (May 2019 through December 2019) i.e., the ‘Multimodal’ order set.

The primary outcome was the overall incidence of inpatient opioid use, defined as opioid intake by the patient. Secondary outcomes included stratification of the incidence of opioid use for the first and second 24-h post-operative periods, as well as the last 24 h of hospitalization; the dose of opioid used, expressed in milligrams of oxycodone (total, first, second, and the last 24 h periods of hospitalization); the incidence of administration and dose of other analgesic medications (acetaminophen and ibuprofen); and incidence of severe pain episodes, defined as pain score ≥7 on a 10 point scale.

We included in our analyses all cesarean deliveries that occurred during the study period. We excluded cases with incomplete records regarding the cesarean delivery, the administered analgesic medications or dosage, or the post-operative pain score assessment, as well as women with a known opioid addiction. In addition, charts with incomplete data or extreme values of pre-pregnancy body mass index (BMI) (≥60 or ≤18.5), charts with missing maternal age, or charts with maternal age ≥50 were excluded as chart reviews indicate they were likely to represent data entry errors or a unique patient population.

Baseline demographic and clinical characteristics such as maternal age, race, ethnicity, pre-pregnancy BMI and weight category, abortion history, parity, and type of cesarean delivery (primary versus repeat) were compared between the two groups as well.

Pearson’s Chi square was used for categorical variables and presented as odds ratios with 95% confidence intervals, while Student *t*-test was used for continuous variables. Differences in the proportions of inpatient opioid administration over time were evaluated with an interrupted time-series analysis using ordinary least-squares (OLS) regression [10]. The interrupted time series is reported using a trend line figure to enable visual comparison of the opioid exposure during the hospital course before and after the implementation of the multimodal order set. For all statistical analyses, significance was set as *p* < 0.05, and the proper assumptions were tested and met. All statistical analyses were conducted using Stata 14.2.

The study received an IRB exemption given the retrospective nature of the analyses and evaluation of de-identified data.

‘Old’ vs. Multimodal post cesarean pain management order sets:

In the United States, it has become a common practice, as it has been at our institution, to have a pre-selected order set for post-cesarean pain management. The ‘old’ post cesarean pain management order set reviewed in this study included a pre-selected standing treatment with ibuprofen 600 mg every 6 h. Oxycodone 5 mg/acetaminophen 325 mg every 3 h for moderate pain (defined as pain score of 4–6), and oxycodone 10 mg/acetaminophen 650 mg every 6 h for severe pain (defined as pain score 7–10) were pre-selected as PRN order (as needed). It did not include a standing order for acetaminophen (Table 1). The new multimodal post cesarean pain management order set differed from the old one as it included pre-selected standing orders for both acetaminophen 975 mg and ibuprofen 600 mg every 6 h, alternating in the times of administration so that patients would receive an analgesic every three hours. In addition, it pre-selected oxycodone immediate release (IR) 5 mg every 3 h for moderate to severe pain (pain score 4–10), and an additional oxycodone IR 5 mg to be administered one hour later if the first dose was ineffective based on pain assessment. Comparison of the two order sets is displayed in Table 1.

In addition, physicians continued to be able to prescribe “a la carte”, individualized, analgesics (narcotic and non-narcotic) based on clinical judgement and patient specific needs. Of note, oxycodone was the only opioid medication used in both the old and multimodal order sets (Table 1).

## 3. Results

**Figure 1:**
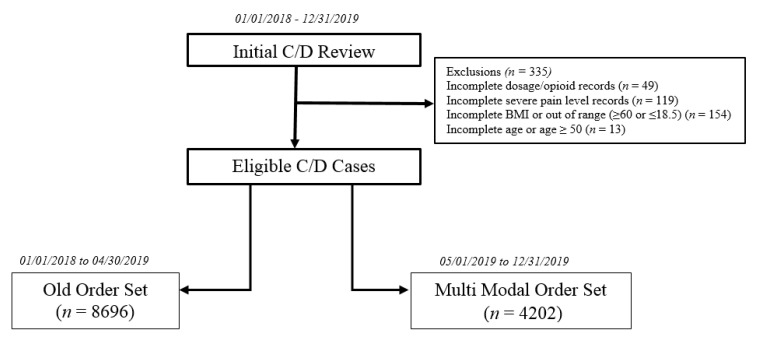
Cesarean Delivery (C/D) Patients Exclusion and Assignment Criteria.

There were 13,233 cesarean deliveries during the study period (January 2018 to December 2019). After excluding 335 women with incomplete data, we assessed a total of 12,898 cesarean births, 8696 of which delivered before the implementation of the multimodal order set (between 1 January 2018, and 30 April 2019). There were 4202 cesarean deliveries in the post implementation period between 1 May 2019, and 31 December 2019 (multimodal order set). Figure 1 displays the study exclusion and assignment criteria. Comparison of baseline demographic and clinical characteristics is displayed in Table 2.

There was a statistically significant difference in the distribution of the specific weight categories between the two groups, although the mean BMI was similar. There were no significant differences in the other demographic and baseline characteristics between the two groups (Table 2). The comparison of the primary and secondary outcomes is displayed in Table 3. The overall incidence of post cesarean opioid use decreased from 67.5% with the old order set to 45.4% with the new multimodal order set (OR 0.4, 95% CI 0.37–0.43; *p* < 0.0001). The mean total dose of oxycodone for patients who received opioids decreased 27%, from 36.6 mg to 26.7 mg (*p* < 0.0001).

**Figure 2:**
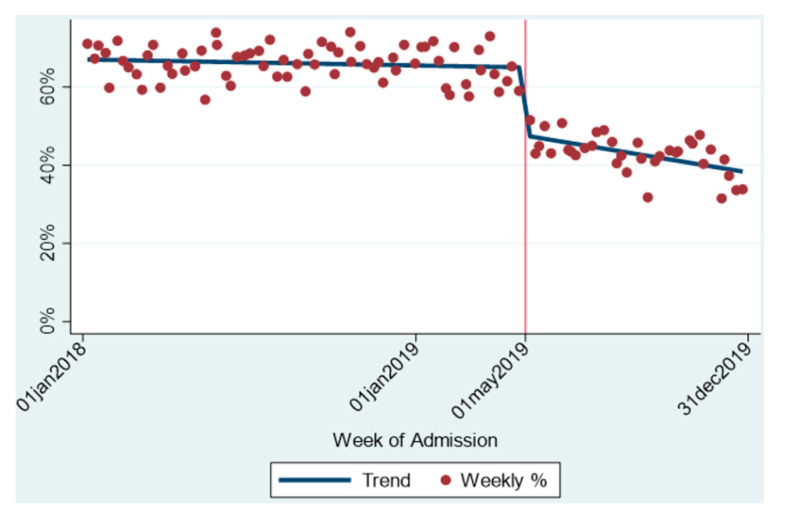
Opioid Exposure during Hospital Course after Cesarean Delivery.

An interrupted time series demonstrating the average percentage of patients that received opioids in the immediate post cesarean recovery throughout the study period is presented in Figure 2. In the initial study period (old order set), the proportion of inpatient post-cesarean opioid usage remained unchanged at approximately 67.5%. Upon implementation of the multimodal order set, an immediate drop was noticed to 45.4% (*p* < 0.001). Furthermore, following this abrupt decrease, a continuous mild reduction in opioid exposure is seen throughout the following eight months since implementation, at a range of 0.25% reduction per week (Figure 2).

Clinically and statistically significant differences were also seen in the incidence of opioid use for each of the 24 h time periods in our stratification: the first 24 h post-operatively (OR 0.475, 95% CI 0.43–0.53); the second 24 h post-operatively (OR 0.42, 95% CI 0.39–0.45); and the final 24 h of hospitalization (OR 0.39, 95% CI 0.36–0.43) (*p* < 0.0001). In the same three time periods, significant opioid dose reduction was seen, with a decrease of 17.2% (from 6.5 mg to 5.3 mg), 23.2% (from 17.6 mg to 13.5 mg), and 20.2% (from 18.8 mg to 15.0 mg), in the first, second, and last 24 h periods, respectively (*p* < 0.0001).

Although the incidence of administration of acetaminophen was high in both groups, and increased only mildly (89.6% and 98.4%, respectively; *p* < 0.0001), the actual dose of acetaminophen increased substantially (from 4563 mg to 8422 mg) in the old and multimodal groups, respectively (*p* < 0.0001). Substantial increases in acetaminophen doses were seen in both patients that were exposed to opioids (increased from 4788 mg to 9133 mg; *p* < 0.0001), as well as in patients that did not take opioids (increased from 4095 mg to 7847 mg; *p* < 0.0001). Significant increase in the dose of acetaminophen were noted for each of the 24 h time periods with dose increase from 802 mg to 1897 mg, from 1355 mg to 2656 mg, and from 1636 mg to 2796 mg, in the first, second, and last 24 h periods, respectively. The difference in ibuprofen dose was statistically but not clinically significant (3815 mg to 4017 mg; *p* < 0.0001).

Severe pain scores decreased from 56.6% with the old order set to 46.3% with the new, multimodal, order set (OR 0.66, 95% CI 0.62–0.71; *p* < 0.0001). Similarly, statistically significant improvements in pain control based on pain scores were further demonstrated during the first and second 24 h post-operative periods, as well as the last 24 h of hospitalization.

## 4. Discussion

After implementing a standardized post-cesarean order set for pain management employing multimodal combination therapy, we found a substantial reduction in both the rate and dose of opioid administration. Post-cesarean opioid use decreased by 32.7%, from 67.5% with the old order set to 45.4% with the multimodal order set (OR 0.4, CI 0.37–0.43, *p* < 0.0001), while the average opioid dose decreased by 27% (36.6 mg to 26.7 mg, respectively, *p* < 0.0001). Of interest, the new, multimodal order set led to improved pain control, with severe pain episodes (pain score ≥ 7) decreasing by 18.2%, from 56.6% with the old order set to 46.3% with the multimodal regimen (OR 0.66, CI 0.62–0.71; *p* < 0.0001).

The primary difference between the old and the new multimodal order sets was the addition of scheduled acetaminophen to the preexisting scheduled NSAID. Unsurprisingly, we found that the acetaminophen dose administered as part of the multimodal order set was significantly higher than that of the old order set for all comparisons analyzed. This highlights the importance of the “around the clock” scheduled administration of both acetaminophen and NSAIDs in order to achieve adequate pain control, translating into less breakthrough pain and lower opioid exposure. This is consistent with smaller studies demonstrating an increase in median acetaminophen milligrams per day with multimodal therapy [6].

Our results are consistent with several small studies, indicating that a multimodal approach to pain control in the immediate post-cesarean period is associated with decreased opioid consumption without increasing hospital stay or median pain scores [6,7,9,11,12,13]. Although all these studies followed similar concepts of scheduled administration of NSAIDs and/or acetaminophen while using opioids for breakthrough pain, the exact protocol may have differed among these studies. A recent quality improvement study, for example, reviewed approximately 570 charts showing that the group who utilized the multimodal approach was associated with a 75% reduction in in-hospital total morphine milligram equivalents without an increase in measured pain or length of hospital stay [6]. Similarly, Rogers et al. recently evaluated the impact of multimodal order set on opioid consumption and pain control in a rather large cohort of patients after vaginal and cesarean deliveries [14]. They reported that opioid use decreased by 26% and 18% among women who delivered vaginally or by cesarean, respectively. Although their cohort included over 14,000 patients, cesarean deliveries accounted for less than a third of the cohort. Interestingly, they reported that the number of patients who reached acceptable pain levels within 24 h was 32% lower with the multimodal order set, making it somewhat less appealing to implement. Our findings, however, suggest that the multimodal order set not only results in decreased opioid consumption, but also in superior pain control for the entire post-operative course and every 24 h period during admission, as noted by frequent pain score assessments.

Studies in other medical disciplines have also examined postoperative opioid prescription patterns with multimodal treatment, providing support for their use. The use of multimodal analgesia was associated with reduced frequency of opioid prescriptions at discharge among 528 patients undergoing thyroid and parathyroid surgery [15]; and a reduction in opioid use and improvement in pain control among patients undergoing penile implant surgery [16]. Implementation of a multimodal analgesic regimen after common arthroscopic procedures, including meniscectomy, rotator cuff repair, and ACL reconstruction, showed a significant decrease in the amount of opioid pain medication prescribed in the postoperative period with the multimodal regimen [17].

A critical aspect in addressing the opioid epidemic is limiting the initial exposure of opioid naïve patients. Studies have shown that the majority of current heroin users were first exposed to prescription opioid medications [18]. Additionally, greater inpatient use of opioids has been associated with increased outpatient opioid consumption [19]. Thus, careful ascertainment of whether a patient requires opioids, and prescribing just enough medication to adequately control the postpartum pain while limiting excess opioids is key. It is imperative for providers and hospitals to come up with models to help assure pain control while limiting opioid use both in the hospital and at home.

The results of this study emphasizes the importance of a user friendly, default, pre-selected order set in the EMR as a key tool facilitating the implementation of pain management protocols. We have shown that using a standardized order set is a feasible and effective approach to implement immediate practice change on a large scale. The decrease in opioid use seen with this order set implementation occurred immediately and was sustained during the subsequent eight months. These findings are consistent with prior research demonstrating that EMRs and their default settings positively affect provider behavior and facilitate adherence to evidence-based guidelines. For example, Anderson et al. showed that the implementation of a standardized, automated thrombo-prophylaxis heparin order for all cesarean deliveries resulted in significant improvement in the rate of deep vein thrombosis prophylaxis [20]. In our experience, a similar approach resulted in a dramatic decrease in opioid use with overwhelming participation by the prescribing providers, making it more broadly applicable and feasible to implement. We believe that preset default pain order sets may be applied across multiple medical, surgical, and obstetrical units to quickly and easily aid in the reduction of opioid exposure, especially in opioid naïve patients.

This study has several strengths. First, our robust database includes detailed, well documented pain management with opioid and non-opioid medications in 12,898 patients across 7 hospitals following cesarean delivery. To our knowledge, this is the largest study to date to demonstrate the benefit of a multimodal pain management regimen after cesarean delivery. Furthermore, we had sufficient power to demonstrate the benefit of the multimodal therapy in decreasing both the exposure to opioids as well as the opioid dosage across several 24 h time periods tested during the patients’ postoperative course. In addition, our database includes detailed documentation of pain score levels that were derived from frequent assessments allowing us to demonstrate the superior pain control obtained with multimodal therapy. The only opioid medication used in both the old and the multimodal order sets was oxycodone, eliminating the potential bias when different type of opioid medications are utilized. Finally, we were able to demonstrate immediate adherence to the new, multimodal, order set following the May 1, 2019, “go live” date in all of our sites, which resulted in an abrupt change in opioid use. These data support the benefits of multimodal therapy but also the important role of EMR-based default order sets as a useful tool to implement such a change.

This study has a few limitations. First, we had no data on post-discharge opioid use, limiting our results to the immediate in-hospital post-operative period. Additionally, we did not evaluate how the change in inpatient opioid ordering habits impacted provider discharge prescribing habits. This is important given that only 19% of the patients in the multimodal group experienced severe pain during the last 24 h of their hospital stay, further suggesting that individualization of opioid prescription upon discharge is needed. A potential follow-up study could aim to investigate the impact of the decreased exposure to opioids in the hospital on both the provider’s opioid prescription habits as well as on patient use at home. Finally, the analysis did not include stratification based on the anesthesia used during surgery. Although not specifically examined in this study, the anesthesia used during both study periods was similar and consisted primarily of regional neuro-axial anesthesia with general anesthesia reserved only for extremely rare cases. The regional neuro-axial anesthesia was mostly spinal with long acting opioid (Duramorph) at a dose of 0.15–0.3 mg. In cases where epidural anesthesia was already administered during labor, the Duramorph dose used was 3–4 mg

## 5. Conclusions

In conclusion, we demonstrated that a standardized post-cesarean order set based on multimodal therapy resulted in substantially decreased exposure to opioids while simultaneously improving pain scores. Embedding such order sets as a default for post-cesarean pain management in the EMR facilitates effective and widespread implementation on a large scale. Hospitals should consider standardizing post-cesarean pain management orders in their EMR to include multimodal combination therapy with acetaminophen and NSAIDS as primary analgesics.

## Figures and Tables

**Table 1:** Old vs. multimodal post cesarean pain management order set.

Pre-Selected	Old Post Cesarean Pain Management Order Set	Multimodal Post Cesarean Pain Management Order Set
Fixed scheduled order	Ibuprofen 600 mg every 6 h No standing order for acetaminophen.	combined scheduled orders Ibuprofen 600 mg every 6 h Acetaminophen 975 mg every 6 h alternating times of administration so that an analgesic is given every three hours
As needed (prn) order	Combination of Oxycodone 5 mg/acetaminophen 325 mg every 3 h for moderate pain (defined as pain score of 4–6) Oxycodone 10 mg/acetaminophen 650 mg every 6 h for severe pain (defined as pain score 7–10)	Oxycodone instant release (IR) 5 mg every 3 h for moderate to severe pain (pain score 4–10), and Additional Oxycodone IR 5 mg to be administered one hour later if the first dose was ineffective (after pain assessment).

**Table 2:** Demographic and Baseline Characteristics.

	Old Order Set (*n* = 8696)	Multimodal Set (*n* = 4202)	*p* ^†^
Age [years, mean (SD)]	33.4 (5.4)	33.4 (5.3)	*p* = 0.626
AMA (≥35 years)	39.3%	39.5%	*p* = 0.749
**Race**			
Non-Hispanic White	51.8%	49.7%	*p* = 0.109
Non-Hispanic Blacks	11.4%	12.1%	
Asian	9.7%	10.1%	
Other, Multi-Racial Groups	27.1%	28.2%	
**Ethnicity**			
Hispanic/Latina	22.2%	22.4%	*p* = 0.115
**BMI [Pre-Pregnancy, mean (SD)]**	26.5 (6·4)	26.6 (6·4)	*p* = 0.368
**Weight Categories (per BMI)**			
Normal (BMI 18.5–24.9)	40.2%	41.3%	*p* < 0.001
Overweight (BMI 25–29.9)	20.8%	21.6%	
Class I Obesity (BMI 30–34.9)	9.8%	11.9%	
Class II Obesity (BMI 35–39.9)	4.4%	4.5%	
Class III Obesity (BMI ≥ 40)	24.7%	20.7%	
**Parity**			
Nulliparity	35.8%	34.8%	*p* = 0.067
Parity 1	28.0%	30.0%	
Multi Parity (≥2)	36.2%	35·2%	
**Prior Abortions**			
None	48.9%	50.0%	*p* = 0.529
One	18.5%	18.3%	
≥Two	32.6%	31.8%	
**Cesarean Delivery**			
Primary	55.0%	56.7%	*p* = 0.100
Repeat	45.0%	43.3%	

† Two-sample *t*-test for continuous variables; x^2^ test of independence for categorical variables. All statistical test assumptions were examined. Categorical percentages may not add to 100 percent due to rounding. AMA—Advanced Maternal Age; BMI—Body mass index.

**Table 3:** Comparison of primary and secondary outcomes between the old and multimodal order set.

	Old Order Set	Multimodal Order Set	Odds Ratio (95% CI)	Sig Level
Patients using Opioids	5872/8696 (67.5%)	1907/4202 (45.4%)	0.40 (0.37–0·43)	*p* < 0.0001
Patients using Opioids (1st 24 h)	1948/8696 (22.4%)	507/4202 (12.1%)	0.48 (0.43–0·53)	*p* < 0.0001
Patients using Opioids (2nd 24 h)	4554/8696 (52.4%)	1318/4202 (31.4%)	0.42 (0.39–0·45)	*p* < 0.0001
Patients using Opioids (last 24 h before discharge)	4430/8696 (50.9%)	1220/4202 (29.0%)	0.39 (0.36–0·43)	*p* < 0.0001

Average dose of Opioids used	36.6 mg	26.7 mg	N/A	*p* < 0.0001
Average dose of Opioids used (1st 24 h)	6.5 mg	5.3 mg	N/A	*p* < 0.0001
Average dose of Opioids used (2nd 24 h)	17.6 mg	13.5 mg	N/A	*p* < 0.0001
Average dose of Opioids used (last 24 h before discharge)	18.8 mg	15.0 mg	N/A	*p* < 0.0001
Any acetaminophen given	89.6%	98.4%	N/A	*p* < 0.0001
Total acetaminophen Administered	Opioids Pos: 4788 mg Opioids Neg: 4095 mg All: 4563 mg	Opioids Pos: 9133 mg Opioids Neg: 7847 mg All: 8422 mg	N/A	*p* < 0.0001
Acetaminophen Administered, first 24 h	802 mg	1897 mg	N/A	*p* < 0.0001
Acetaminophen Administered, second 24 h	1355 mg	2656 mg	N/A	*p* < 0.0001
Acetaminophen Administered, last 24 h	1636 mg	2796 mg	N/A	*p* < 0.0001
Any ibuprofen administered	94·7%	95·40	N/A	*p* = 0.095
Total Ibuprofen Administered	3815 mg	4017 mg	N/A	*p* < 0.0001
Severe pain (score ≥ 7)	4923/8696 (56.6%)	1947/4202 (46.3%)	0.66 (0.62–0.71)	*p* < 0.0001
Severe pain (1st 24 h post op)	2460/8696 (28.3%)	876/4202 (20.8%)	0.67 (0.12–0.73)	*p* < 0.0001
Severe pain (2nd 24 h post op)	2986/8696 (34.3%)	1019/3183 (24.3%)	0.61 (0.56–0.66)	*p* < 0.0001
Severe pain (last 24 h before discharge)	2482/8696 (28.5%)	799/4202 (19.0%)	0.59 (0.54–0.64)	*p* < 0.0001
